# The dysbiosis of ovine foot microbiome during the development and treatment of contagious ovine digital dermatitis

**DOI:** 10.1186/s42523-021-00078-4

**Published:** 2021-02-17

**Authors:** J. S. Duncan, J. W. Angell, P. Richards, L. Lenzi, G. J. Staton, D. Grove-White, S. Clegg, G. Oikonomou, S. D. Carter, N. J. Evans

**Affiliations:** 1 Department of Livestock and One Health, Institute of Infection, Veterinary and Ecological Science, University of Liverpool, Leahurst Campus, Neston, Wirral CH64 7TE UK; 2 Wern Veterinary Surgeons, Department of Research and Innovation, Unit 11, Lon Parcwr Industrial Estate, Ruthin, LL15 1NJ UK; 3 Department of Veterinary Pathology, Institute of Infection, Veterinary and Ecological Science, University of Liverpool, Leahurst Campus, Neston, Wirral CH64 7TE UK; 4 Centre for Genomic Research, Department of Evolution, Ecology and Behaviour, Institute of Infection, Veterinary and Ecological Science, University of Liverpool, Liverpool, L69 7ZB UK; 5 Department of Infection Biology & Microbiomes, Institute of Infection, Veterinary and Ecological Science, The University of Liverpool, Leahurst Campus, Neston, Wirral CH64 7TE UK; 6 School of Life Sciences, College of Science, University of Lincoln, Brayford Pool Campus, Lincoln, LN6 7TS UK

**Keywords:** Sheep, Lameness, CODD, Footrot, Microbiome

## Abstract

**Background:**

Contagious Ovine Digital Dermatitis (CODD) is an emerging and common infectious foot disease of sheep which causes severe welfare and economic problems for the sheep industry. The aetiology of the disease is not fully understood and control of the disease is problematic. The aim of this study was to investigate the polybacterial aetiopathogenesis of CODD and the effects of antibiotic treatment, in a longitudinal study of an experimentally induced disease outbreak using a 16S rRNA gene amplicon sequencing approach.

**Results:**

CODD was induced in 15/30 experimental sheep. During the development of CODD three distinct phenotypic lesion stages were observed. These were an initial interdigital dermatitis (ID) lesion, followed by a footrot (FR) lesion, then finally a CODD lesion. Distinct microbiota were observed for each lesion in terms of microbial diversity, clustering and composition. *Porphyromonadaceae, Family XI, Veillonellaceae* and *Fusobacteriaceae* were significantly associated with the diseased feet. *Veillonellaceae* and *Fusobacteriaceae* were most associated with the earlier stages of ID and footrot rather than CODD. Following antibiotic treatment of the sheep, the foot microbiota showed a strong tendency to return to the composition of the healthy state. The microbiota composition of CODD lesions collected by swab and biopsy methods were different. In particular, the *Spirochaetaceae* family were more abundant in samples collected by the biopsy method, suggesting that these bacteria are present in deeper tissues of the diseased foot.

**Conclusion:**

In this study, CODD presented as part of a spectrum of poly-bacterial foot disease strongly associated with bacterial families *Porphyromonadaceae, Family XI* (a family in Clostridiales also known as Clostridium cluster XI)*, Veillonellaceae* and *Fusobacteriaceae* which are predominately Gram-negative anaerobes. Following antibiotic treatment, the microbiome showed a strong tendency to return to the composition of the healthy state. The composition of the healthy foot microbiome does not influence susceptibility to CODD.

Based on the data presented here and that CODD appears to be the severest end stage of sheep infectious foot disease lesions, better control of the initial ID and FR lesions would enable better control of CODD and enable better animal welfare.

**Supplementary Information:**

The online version contains supplementary material available at 10.1186/s42523-021-00078-4.

## Introduction

Contagious ovine digital dermatitis (CODD) is an emerging, infectious foot disease of sheep first reported in the UK in 1997 [1]. Epidemiological surveys suggest that CODD now occurs on approximately 50% of UK farms [2, 3], exhibiting a within farm prevalence of between 2 and 50% [3] and has now also been reported in Ireland [4], Germany [5] and Sweden (personal communication) with a very similar manifestation has now appeared in UK goats [6] and wild American elk [7]. The clinical presentation of CODD is an inflammatory lesion on the dorsal coronary band which develops into progressive underrunning of the hoof horn in a distal direction, eventually resulting in avulsion of the entire hoof capsule [8]. In animal welfare terms, CODD is the most severe form of sheep lameness [9]. Control relies heavily on antibiotic treatments, in particular the macrolides which are categorized by the World Health Organization as a highest priority - critically important antimicrobials, essential for human health. Therefore, due to the animal welfare and public health impact, CODD emergence has been identified as a priority disease issue for the sheep industry [10]. The development of non-antibiotic control strategies such as vaccination and evidenced based biosecurity protocols is crucial and requires a greater understanding of CODD aetiopathogenesis.

Spirochetal bacteria are associated with CODD lesions, specifically three members of the *Treponema* genus, namely bacteria of the *Treponema medium* phylogroup, *Treponema phagedenis* phylogroup and *Treponema pedis* [11, 12]. These bacteria are also considered causal in bovine digital dermatitis (DD) [13], leading to the suggestion that DD treponemes may have crossed species from cattle to sheep to cause CODD.

However, CODD also shares substantial bacteriological and epidemiological features with footrot, a common sheep foot disease that is endemic in many countries worldwide. Studies of CODD affected feet identified the presence of the causal agents of footrot, *Dichelobacter nodosus* and *Fusobacterium necrophorum* [11]. In addition, key risk factors for both diseases are remarkably similar with congruence acknowledged for wet underfoot conditions, poor biosecurity practices, foot trimming and flock size [14, 15]. Footrot presence on farms and failure to apply footrot control measures such as vaccination and/or prompt individual treatment are also strongly associated with CODD occurrence [2, 14, 15]. Therefore, current evidence on CODD aetiology requires clarification as to whether CODD is an entirely novel infectious foot disease or whether it results from secondary invasion of pre-existing footrot lesions. Furthermore, dissection of the precise roles of the different bacteria within CODD lesions during lesion development is needed and can help in the development of better treatment and control strategies.

In order to unravel the aetiology of CODD we assessed clinical lesion development and changes in the bacterial communities of sheep’s feet during transition from healthy to CODD diseased state. To ensure accurate implication of responsible pathogens this study was novel in investigating a naturally occurring outbreak of disease in a previously CODD naïve flock in a controlled experimental environment.

The objectives of the current study were to:- 1) Describe the clinical, phenotypic changes in the ovine foot during the development of CODD, 2) Describe the changes in the microbiome of the ovine foot during the development of CODD, 3) Describe the changes in the microbiome of the ovine foot following antibiotic treatment of CODD, 4) Determine whether differences in the healthy ovine foot microbiome predict susceptibility to CODD, 5) Compare the ovine foot microbiome of CODD lesions obtained by different lesion sampling methods.

## Results

### Quality control and sequencing results

The number of reads per sample are summarised in Supplementary file 2. One DNA extraction negative control was amplified and sequenced, producing less than100 reads. As this was considerably fewer reads than the proccessed samples, the degree of contamination during DNA extraction was considered negligble and these sequences were not removed prior to analysis. Negative controls included during PCR steps indicated no contamination had occurred at this stage. One hundred fifty-six samples were included in the data set and the number of reads per sample was variable within and between sample types. The median number of reads per sample was 217,628 (Interquartile range, IQR: 103,638). A total of 16,177 different ASVs were identified and taxonomically assigned. The median number of ASVs per sample was 120,736 (IQR 48,811). Taxonomic analysis was primarily carried out at family level to minimise information lost due to unclassified samples at genera and species level.

### Clinical description of CODD lesion development

Only CODD lesions that progressed past CODD stage 2 [8] during the experimental study were included so as to avoid confusion with non-specific injuries to the coronary band of the foot that can be confused with CODD grade 1 lesions. Applying this case definition, 15 of the 30 sheep (50%) and 26 of the 120 ft (21.67%) in the study developed CODD lesions.

**Fig. 1:**
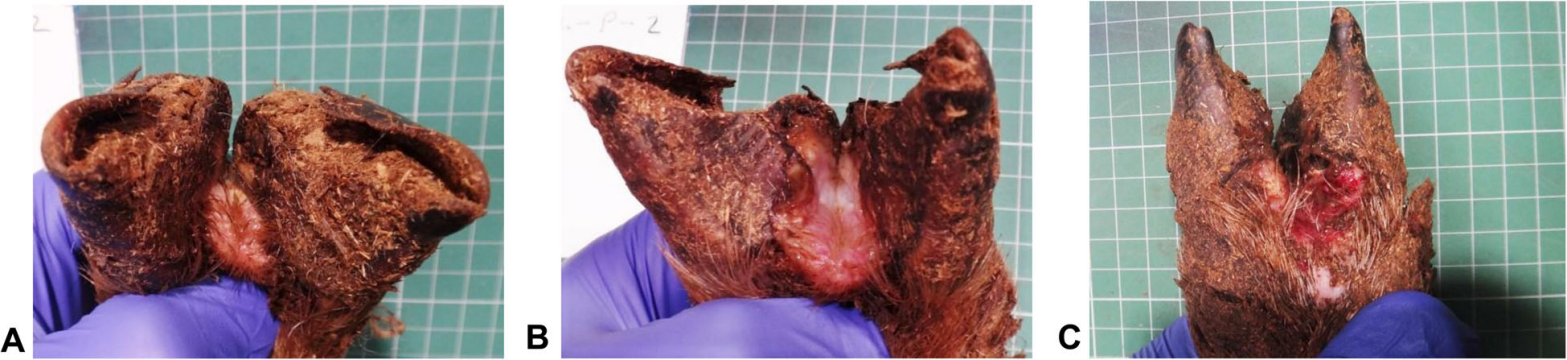
Sheep foot lesions observed during the development of CODD. **a** Interdigital dermatitis lesion, **b** footrot lesion, **c** CODD lesion

All these CODD lesions were observed to follow a specific clinical pattern of lesion development consistent with the descriptions of footrot lesions as described by Egerton et al. [16] and CODD lesions as described by Angel et al. [8] . The disease process began as initial interdigital dermatitis (ID), whereby the interdigital skin was inflamed (Fig. 1a). This was the followed by the footrot (FR) stage with progressive underrunning of horn of the hoof beginning at the axial margin and extending across the sole (Fig. 1b). Finally, at the CODD stage, an inflammatory lesion would be present at the dorsal coronary band which would progress to separate the hoof horn capsule from the underlying dermis in a ventral direction (Fig. 1c). The median survival time for a sheep to develop an ID lesion was 88 days, FR lesion 103 days and CODD lesion was 116 days from the start of the experiment.

### Changes in the sheep foot microbiome during the development of CODD

Foot swab samples from 6 sheep in the study whose feet developed typical, progressive CODD lesions were selected for the analysis of changes in foot microbiome composition during the development of CODD lesions. These sheep were selected on the basis that the foot had undergone the previously described clinical progression, namely from a healthy foot to interdigital dermatitis to footrot and then to CODD. Samples included were those from: Sheep 2 (*n* = 16 samples), sheep 5 (*n* = 22 samples), sheep 8 (*n* = 17 samples), sheep 9 (*n* = 15 samples), sheep 12 (*n* = 19 samples) and sheep 14 (*n* = 16 samples). As CODD developed at different rates and a different time points in the study for each sheep, a different number of samples for each disease stage was available for each sheep, providing 105 samples. Based on the associated clinical metadata, these samples were classed as A_Healthy (samples collected from healthy feet at least 15 days prior to lesion development) (*n* = 23); B_Healthy, (samples collected from healthy feet at 0–14 days prior to lesion development)(*n* = 18); C_ID (samples collected from feet classed as affected by interdigital dermatitis)(*n* = 20); D_Footrot, (samples collected from sheep’s feet classed as affected by footrot) (*n* = 20); E_CODD (samples collected from sheep’s feet classed as affected by CODD) (*n* = 24).

#### Changes in bacterial diversity of sheep foot microbiome (alpha and beta) during the development of CODD

**Fig. 2:**
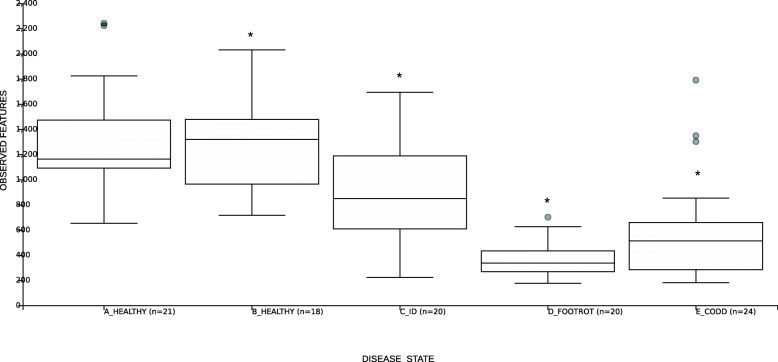
Box and whisker plots of alpha diversity as measured by observed ASVs for the different stages of CODD lesion development. * represents *p* < 0.05

Changes in microbial community diversity for disease stages observed during the development of the CODD lesions were determined by examining the number of observed ASVs at each stage. Generally, when compared with the healthy state (up to 2 weeks before disease onset), as CODD developed there was a reduction in the number of observed ASVs, consistent with dominance of disease associated bacterial species in the lesions across all foot disease states (*p* < 0.05) (Fig. 2). Pairwise comparisons were then used to identify differences in diversity between the different disease states observed as the clinical lesions progressed. Significant reductions in ASV numbers (*p* < 0.05) were observed when the foot moved from the B_ Healthy state (2 weeks before disease onset) to the ID stage and then to the FR stage. However, there was no difference in number of ASVs between the FR and CODD stages (Supplementary Table 1).

**Fig. 3:**
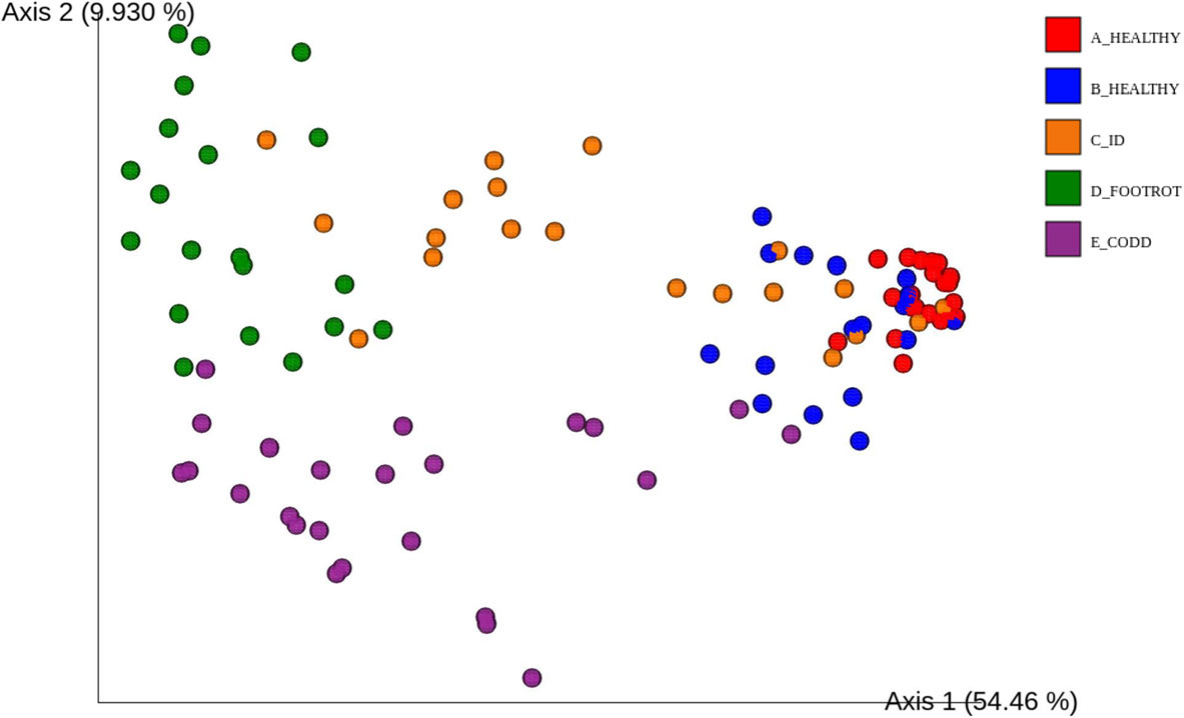
Principle Coordinate Analysis (PCoA) plot showing differences in weighted UniFrac distances at different stages of CODD lesion development. ANOSIM for disease state overall (ANOSIM R statistic =0.64329, *p* = 0.001). Pairwise ANOSIM tests between microbiota at each phenotypic lesion stage (A_Healthy, B_Healthy, C_ID, D_Footrot, E_CODD) (*p* = 0.001)

Distinct clustering patterns of microbiota for each phenotypic stage observed in the development of CODD lesions were also observed in the beta diversity analysis (Fig. 3). When measured with a weighted UniFrac metric, beta diversity in terms of location and spread was significantly affected by disease state overall (ANOSIM R statistic =0.64329, *p* = 0.001). Furthermore, pairwise ANOSIM tests demonstrated significant differences (*p* = 0.001) between microbiota at each phenotypic lesion stage of CODD development, in terms of microbiota location, dispersion and correlation structure (Supplementary Table 2).

#### Changes in composition of sheep foot microbiome during the development of CODD (Gneiss analysis)

1. *Identification of Balances Significantly Associated with Changes in Disease State*

**Fig. 4:**
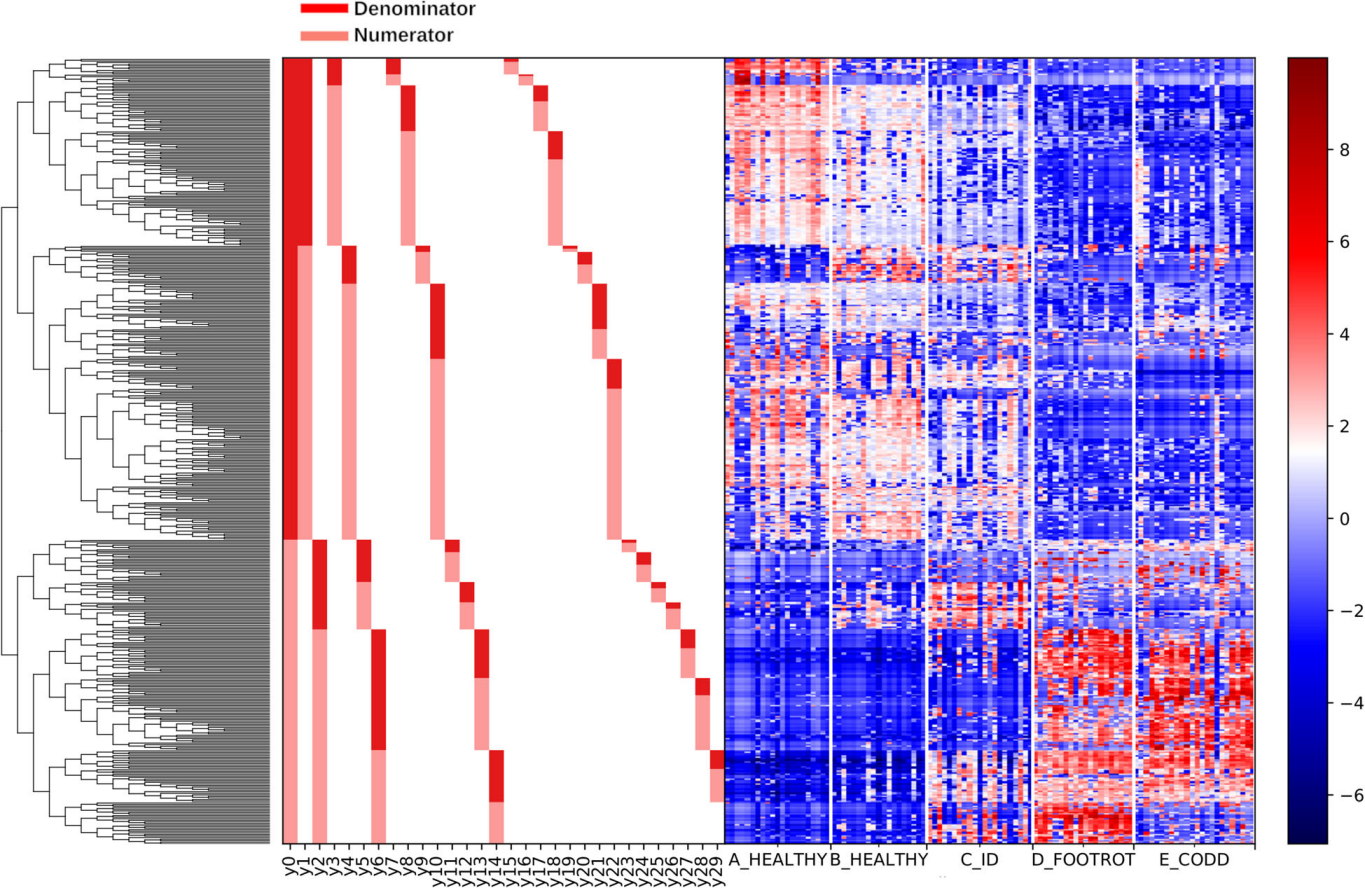
A dendogram heatmap showing log_10_ abundance of ASVs in the foot microbiota at different stages of CODD lesion development

**Fig. 5:**
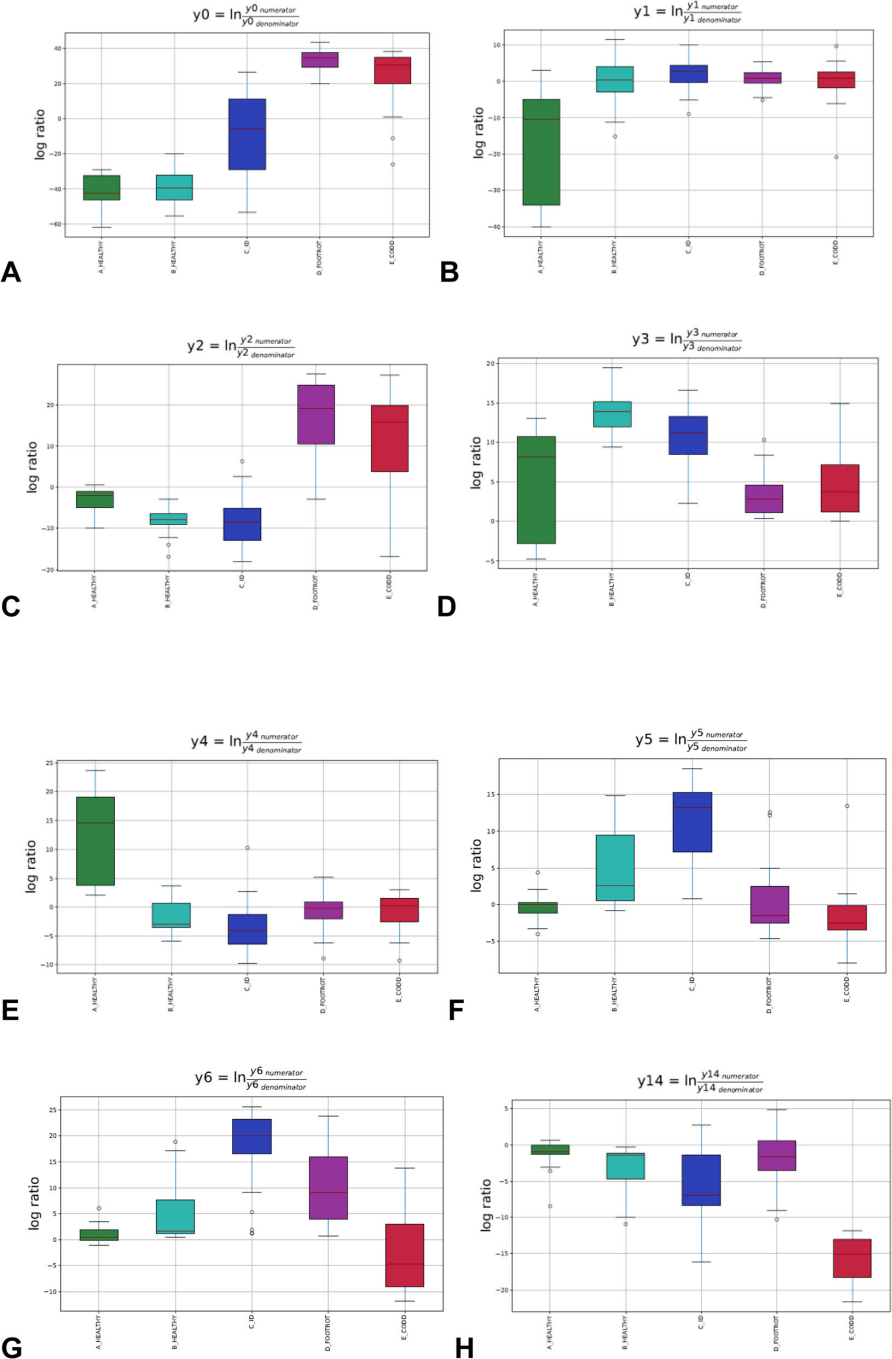
Gneiss analysis log_10_ ratio balances significantly different (*p* < 0.05) between stages of CODD lesion development. **a** balance y0, **b** balance y1, **c** balance y2, **d** balance y3, **e** balance y4(**a**), **f** balance y5, **g** balance y6, **h** balance y14. A lower log_10_ ratio shows a shift in the balance toward denominator ASVs whilst a higher log_10_ ratio shows a shift towards numerator ASVs as visually represented in Fig. 4

The relative abundance of bacterial taxa in microbiota of sheep’s feet at different stages of CODD lesion development differed. The overall linear regression model fit was R^2^ = 0.503. B_Healthy samples accounted for 3.31% of variance with ID, Footrot and CODD samples accounting for 8.44, 23.8 and 20.4% of variance respectively. Investigation of balance log_10_ ratios which were significantly different (*p* < 0.05) between disease states and inspection of the dendogram heatmap (Figs. 4 and 5) allow a description of the significant changes in microbiome composition as CODD developed.

Inspection of the dendogram heat map and examination of log_10_ ratio of balances clearly show y0 was significantly lower in A_Healthy than ID (β = 29.9, *p* < 0.001), Footrot (β = 74.0, *p* < 0.001) and CODD (β = 63.8, *p* < 0.001) samples (Fig. 5a) but was not significantly different from B_Healthy samples (β = 1.98, *p* = 0.9). The dendogram heatmap shows that broadly, y0_denominator_ ASVs were more abundant in healthy samples while y0_numerator_ were more abundant in diseased samples with ID samples acting as an intermediate.

Subdivisions of y0_denominator_ ASVs reveal further differences between A_Healthy, B_Healthy and ID samples; these stages represent the earlier stages of the CODD disease development process and therefore examination of microbial compositional changes here give an indication of earlier changes in the bacterial community. The log_10_ ratio of balance y1 was significantly lower in A_Healthy samples compared to B_Healthy (β = 16.5, *p* < 0.001) and ID (β = 18.6, *p* < 0.001) samples (Fig. 5b). The dendogram heatmap shows that this was due to a higher abundance of some y1_denominator_ ASVs in A_Healthy samples, specifically those further identified by balance y3_denominator_ (Fig. 5d). In addition, there was a higher abundance of some y1_numerator_ ASVs in B_Healthy and ID samples, the most abundant taxa identified by y4_denominator_ (Fig. 5e).

Balance y2 (Fig. 5c) is useful to describe differences in microbial composition as the foot lesions develop from ID stage as this balance was significantly lower in ID samples compared to Footrot (t = 10.25, *p* < 0.001) and CODD (t = 6.4, *p* < 0.001) samples. The dendogram heatmap shows that this was due to a higher abundance of some y2_denominator_ ASVs in ID samples, specifically y5_numerator_ ASVs as demonstrated by a significantly higher log_10_ ratio in ID samples when compared to other groups (A_Healthy: t = 8.34, *p* < 0.001; B_Healthy: t = 3.25, *p* = 0.002; Footrot: t = 6.12, *p* < 0.001; CODD: t = 8.40, *p* < 0.001; Fig. 5f).

Furthermore, balance y6 log_10_ ratio was significantly higher in ID samples compared to Footrot (t = 3.16, *p* = 0.003) and CODD (t = 8.17, *p* < 0.001) due to a higher abundance of y6_denomiantor_ ASVs in footrot and CODD samples (Fig. 5g).

Differences in microbiome composition between footrot and CODD are observed in balance y14 whose log_10_ ratio was significantly higher in ID and footrot samples compared to CODD samples (t = 7.96, *p* < 0.001 and t = 13.3, *p* < 0.001 respectively) due to an increased abundance of y14_numerator_ ASVs in these samples (Fig. 5h).

2) *Bacterial Taxa Associations with Changes in Disease State*

The Gneiss analysis results were then used to classify the ASVs into four groups as follows:

1. **Healthy ASVs** - ASVs with higher abundance in A_Healthy samples (y3_denominator_) and ASVs with higher abundance in A_Healthy, B_Healthy and ID samples (y3_numerator_ and y4_numerator_);
2. **Intermediate ASVs** - ASVs with higher abundance in B_Healthy and ID samples (y4_denominator_) and ASVs with higher abundance in ID samples (y5_numerator_);
3. **Diseased ASVs** - ASVs with higher abundance in ID and Footrot samples (y14_numerator_), ASVs with higher abundance in ID, Footrot and CODD samples (y14_denominator_) and ASVs with higher abundance in Footrot and CODD samples (y6_denominator_ and y11_denominator_);
4. **ASVs not differentially abundant between sample groups** (y11_numerator_).

**Fig. 6:**
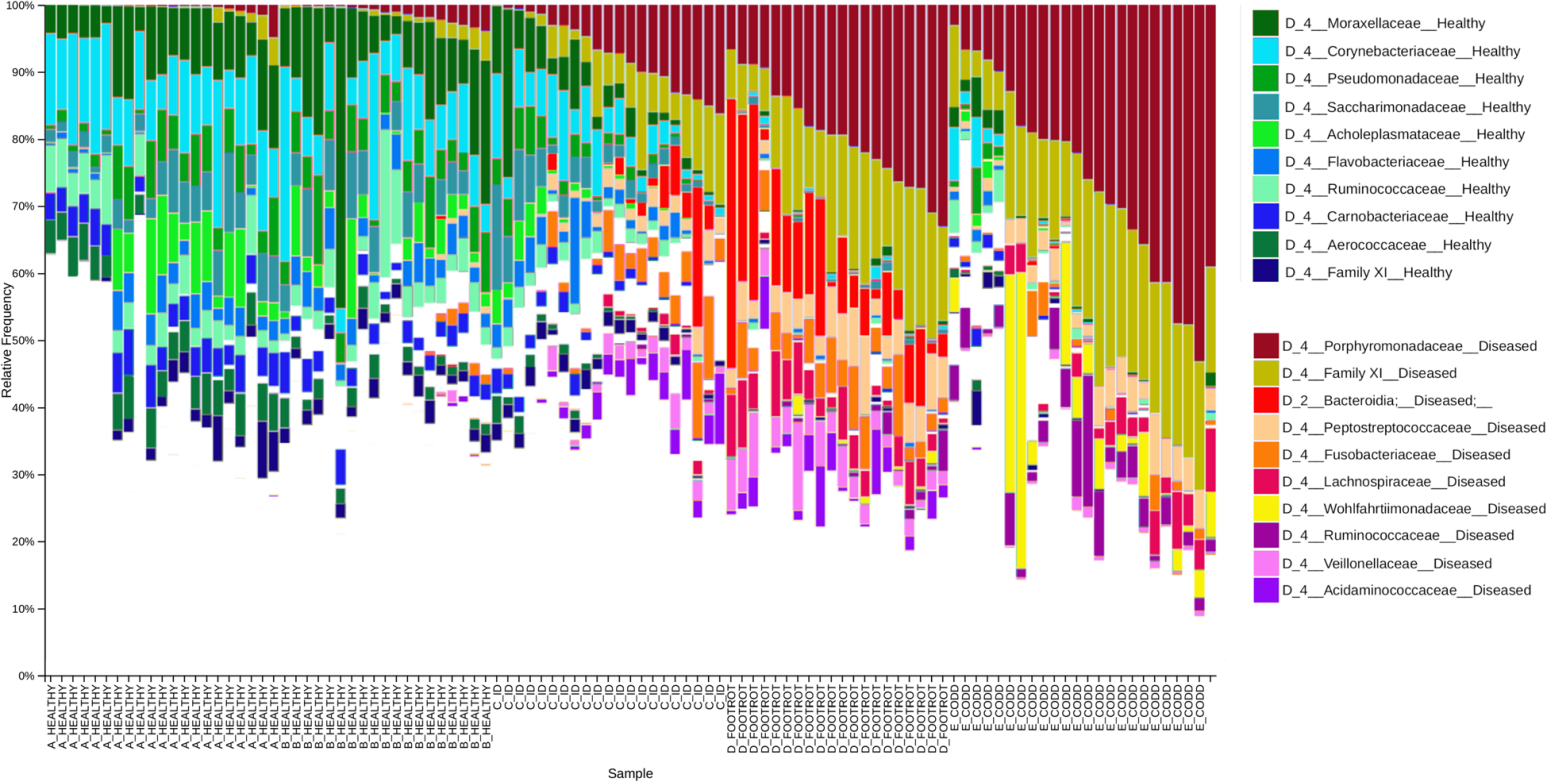
A taxa plot showing relative abundance of the 10 most abundant bacterial families (identified from the Healthy and Diseased ASV groups) in the microbiomes of the different diseased states (A_Healthy, B_Healthy, C_ID, D_Footrot and E_CODD)

The families of ASVs identified by Gneiss as more abundant between disease states were plotted in a taxa bar plot to compare the relative abundance of the most abundant 10 families from both the Healthy and Diseased ASV groups (Fig. 6). The most abundant families in the healthy group were *Moraxellaceae, Corynebacteriaceae Pseudomonaceae Saccharimonadaceae Acholeplasmataceae, Flavobacteriaceae, Ruminococcaceae Carnobacteriaceae Aerococcaceae, Family XI.* Whilst in the diseased sheep the most abundant 10 families were *Porphyromonadaceae, Family XI, Bacterioida Peptostreptococcaceae Fusobacteriaceae Lachnospiraceae, Wohlfahrtiimonadaceae, Ruminococcaceae Veillonellaceae, Acidaminococcaceae.*

Examination of the distribution of ASVs in these families between Healthy, Intermediate and Diseased ASV groups demonstrated significant associations (*p* < 0.05) between specific taxa, namely *Porphyromonadaceae, Family XI, Veillonellaceae* and *Fusobacteriaceae* and the Diseased ASV group (Table 1). Furthermore, the taxa plot shows the relative abundance of these four families increasing from Healthy samples to ID, footrot and CODD samples (Fig. 6), although an increased abundance of *Veillonellaceae* and *Fusobacteriaceae* was most associated with ID and footrot rather than CODD.

**Table 1:**
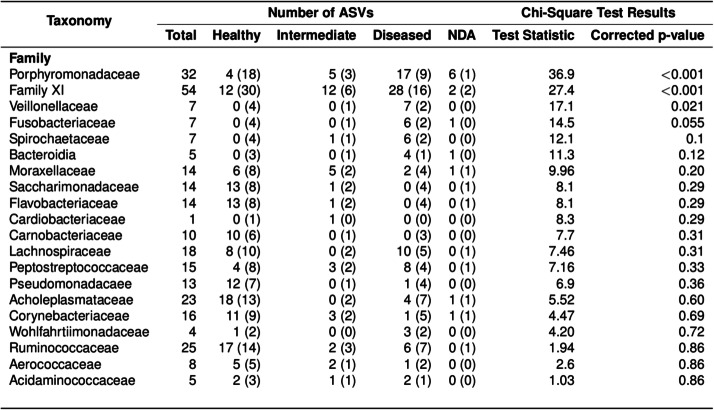
Taxonomic divisions containing ASVs identified as differentially abundant between Healthy, Intermediate and Diseased feet. Families which had either a significantly different distribution to the expected value or were most associated with one ASV group are shown. Expected distributions are displayed in brackets

No other families were significantly differently distributed between Healthy, Intermediate and Diseased groups. This may be an artefact of small numbers of ASVs assigned to these families. For example, *Bacteroidia* was not identified as significantly differently distributed between groups even though 4 of 5 ASVs were assigned to the Diseased ASV group. The taxa plot (Fig. 6) shows that the relative abundance of *Bacteroidia* was especially increased in footrot samples. Equally, the majority of ASVs assigned to *Corynebacterium, Pseudomonadaceae, Saccharimonadaceae, Acholeplasmataceae, Flavobacteriaceae, Carnobacteriaceae* and *Aerococcaceae* were assigned to the Healthy ASV group with these families featuring prominently in the taxa plots of Healthy samples. *Ruminococcaceae* and *Lachnospiraceae*, families commonly associated with the intestinal microbiome, were more divided between Healthy and Diseased ASV groups. The taxa plot shows that a different profile of *Ruminococcaceae* and *Lachnospiraceae* was found in Healthy samples compared to footrot and CODD samples.

Pathogens previously identified as important in the pathogenesis of ID, footrot and CODD were found within the dataset. One ASV assigned to *Dichelobacter nodosus* was classified as an Intermediate ASV and was found mainly in ID samples. Three ASVs assigned to *Fusobacterium necrophorum* were classified as Diseased ASVs and were found mainly in ID and footrot samples. Six ASVs were identified as *Treponema* with all six classified as Diseased ASVs which were present in ID and footrot samples. When these six treponeme sequences were compared with a wide range of relevant, previously isolated treponeme 16S rRNA gene sequences [17], three of the sequences had 100% sequence identity to the key DD associated treponemes, specifically *Treponema medium-*phylogroup strain T19, *Treponema phagedenis* phylogroup strain T320A and *Treponema pedis* strain T3552B^T^. The further three treponeme sequences identified all shared 98.4–99.6% nucleotide sequence identity with the *Treponema medium* phylogroup strain T19.

### Changes in the sheep foot microbiome following antibiotic treatment of CODD

Foot swab samples from five sheep in the study whose feet developed progressive CODD lesions and were subsequently treated (2 doses of long acting amoxicillin every 48 h), were selected for the analysis of changes in foot microbiome composition following treatment. Antibiotic treatment of sheep was effective in achieving a clinical cure (CODD lesion stage grade 5 [8]) in all sheep within 7 days of initial treatment. Based on clinical presentation foot lesions were classified as A_Healthy (samples collected from sheep feet classed as healthy upon entry to study (*n* = 5)); B_CODD (samples collected from sheep’s feet clinically assessed as having active CODD lesions (*n* = 21)); C_Treat (samples collected from sheep’s feet 2 weeks post treatment and classed as healed, grade 5 CODD lesions (*n* = 5)).

#### Changes in bacterial diversity of sheep foot microbiome (alpha and beta) following treatment of CODD

**Fig. 7:**
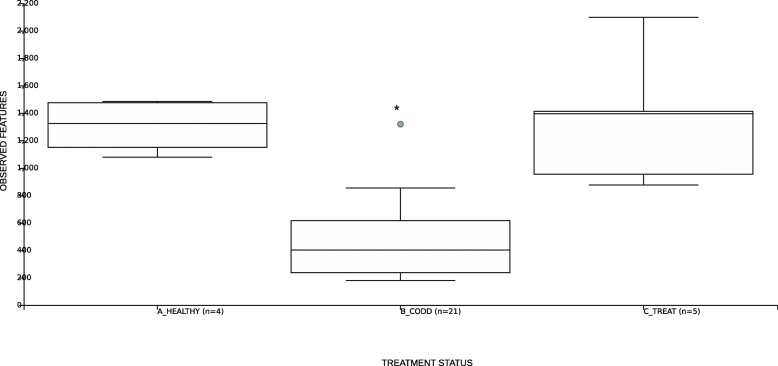
Box and whisker plots of alpha diversity as measured by observed ASVs for samples taken from healthy (A_Healthy), CODD affected (B_CODD), antibiotic treated (C_Treated) sheep’s feet

Changes in microbial community diversity for A_Healthy, B_CODD and C_Treated feet were measured by examining the number of observed ASVs (Fig. 7). Pairwise comparisons found significant reductions in observed ASV numbers between B_CODD feet and both A_Healthy feet (*p* < 0.01) and C_Treated feet (*p* < 0.001). However, no differences were observed in diversity of samples from A_Healthy feet and C_Treated feet (*p* = 0.62) indicating similar bacterial diversity in the microbiomes of the healthy and treated feet (Supplementary Table 3).

**Fig. 8:**
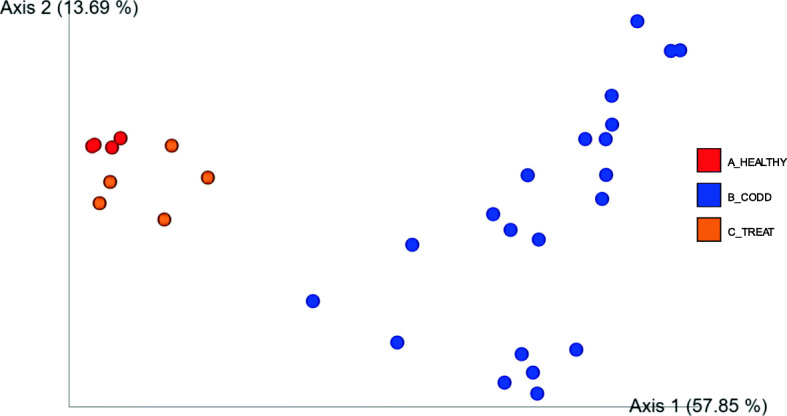
Principle Coordinate Analysis (PCoA) plot showing differences in weighted UniFrac differences of samples from healthy (A_Healthy), CODD affected (B_CODD), antibiotic treated (C_Treated) sheep’s feet. ANOSIM tests show beta diversity was significantly different overall three states (R test statistic 0.825846, *p* = 0.001), Pairwise ANOSIM tests between microbiota at each lesion type (A_Healthy, B_CODD, C_Treated (*p* < 0.05)

Similarly, when measured with a weighted UniFrac metric, the ANOSIM tests show beta diversity was significantly different between all three groups, B_CODD and both A_Healthy, C_Treated feet (R test statistic 0.825846, *p* = 0.001). Furthermore Pairwise ANOSIM tests between microbiota at each lesion type (A_Healthy, B_CODD, C_Treated (*p* < 0.05). (Supplementary Table 4). However, the PCoA plot shows that the A_Healthy and C_Treated feet tend to cluster together suggesting that following treatment the community clustering tends to return towards the healthy state (Fig. 8).

#### Changes in composition of sheep foot microbiome following treatment of CODD (Gneiss analysis)

1. *Identification of Balances Significantly Associated with Changes in Disease State*

**Fig. 9:**
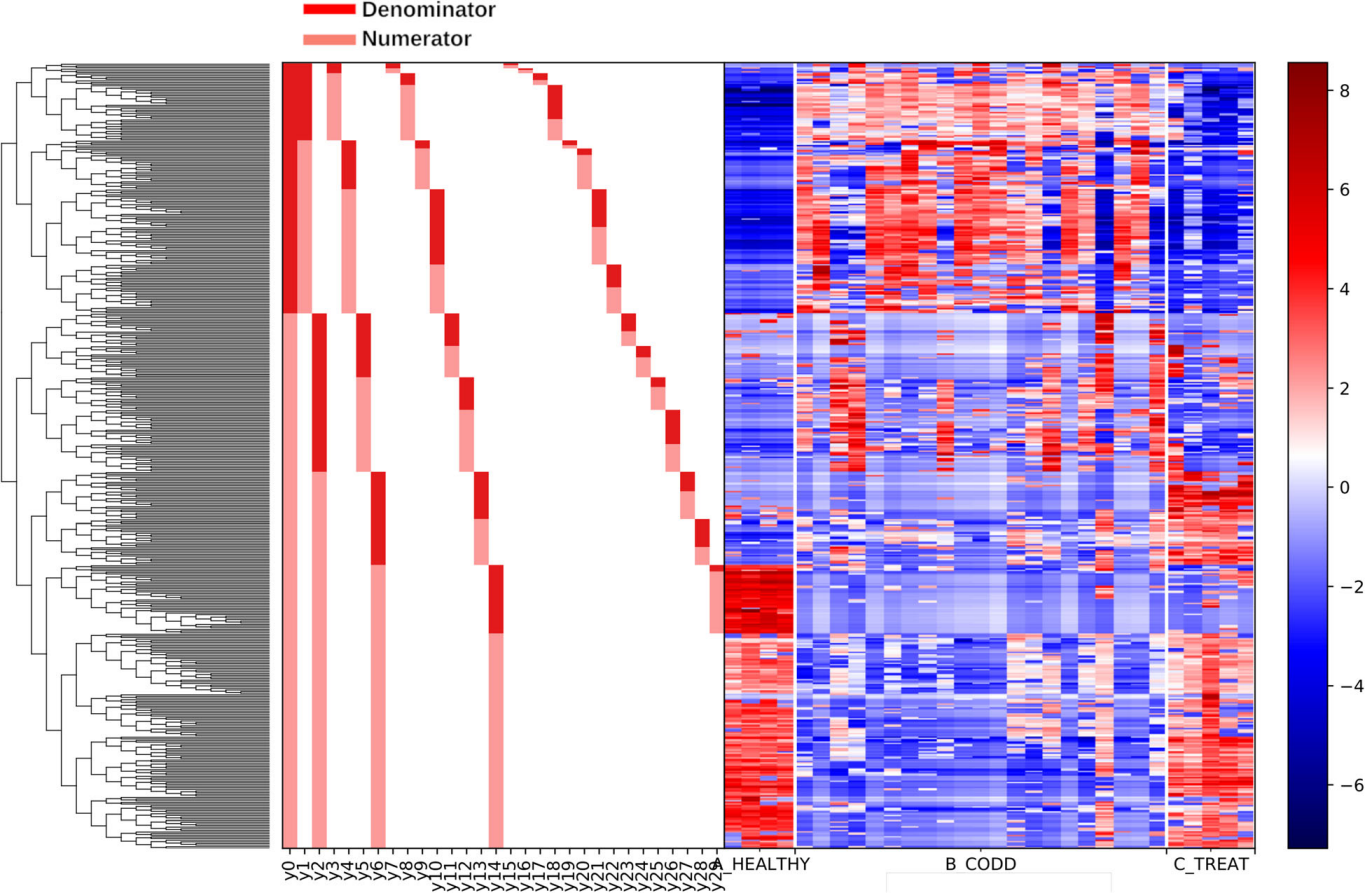
Dendogram heatmap showing log_10_ abundance of ASV in the foot microbiota of samples from healthy (A_Healthy), CODD affected (B_CODD) and antibiotic treated (C_Treated) sheep’s feet

**Fig. 10:**
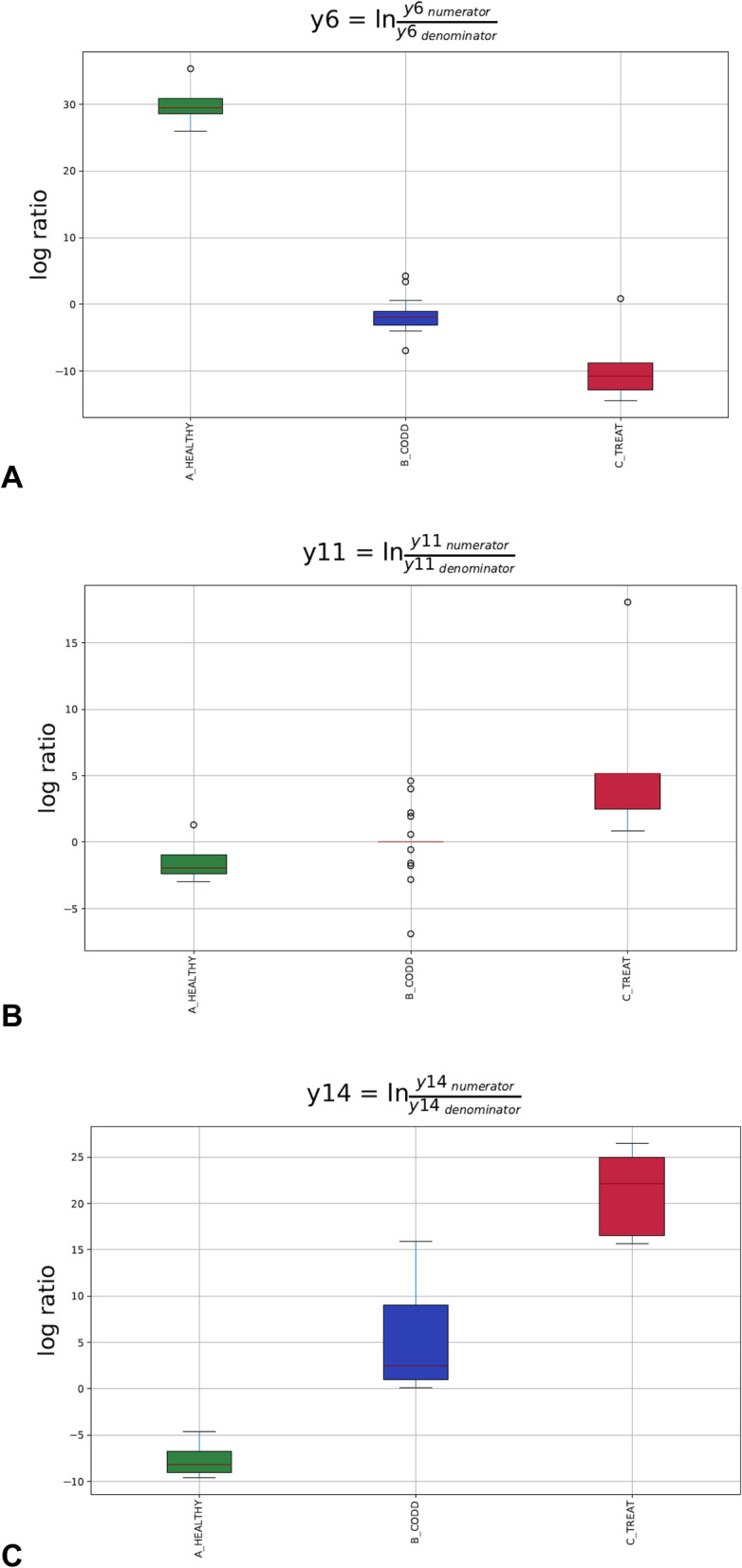
Gneiss analysis Log_10_ ratio balances whose log_10_ ratio balance was significantly different (*p* < 0.05) between microbiomes of samples taken from healthy (A_Healthy), CODD affected (B_CODD) and antibiotic treated (C_Treated) sheep’s feet. A lower log_10_ ratio shows a shift in the balance toward denominator ASVs whilst a higher log_10_ ratio shows a shift towards numerator ASVs as visually represented in Fig. 9

The overall linear regression model fit was R^2^ = 0.468 with covariate “Treatment” as the only variable. B_CODD accounted for 17.44% of variance with C_Treat samples accounting for 37.83% of variance. The dendogram heat map (Fig. 9), in conjunction with balance analysis demonstrates a clear pattern of microbiome composition change as foot lesions move from healthy to CODD state and then healed state. There was a strong tendency for the microbiome of the healed state to return to that of the healthy microbiome. However, 3 log_10_ ratio balances y6 (B = -39.18, *p* < 0.001), y11(B = 7.74, *p* = 0.004) and y14 (B = 28.78, *P* < 0.001) showed significant differences in log_10_ ratios between the groups (Fig. 10a-c).

2) *Bacterial Taxa Associations with Changes in Disease State*

The log_10_ ratio of balance y6 (Fig. 10a) was significantly lower in C_Treated feet compared with the healthy feet due to higher abundance of some y6 denominator ASV in the treated feet compared to healthy feet. These ASVs were mainly assigned to *Ruminococcaceae, Lachnospiraceae, Carnobacteriaceae, Staphylococcaceae, Pseudomonadaceae.* The log_10_ ratio balance of y11 (Fig. 10b) was significantly higher in C_Treated feet compared to the healthy state due to higher abundance of some y11 numerator ASVs which were assigned to Family XI, *Corynebacteriaceae, Acholeplasmataceae, Staphylococcaceae, and Rhodobacteraceae*. The log_10_ ratio balance of y14 (Fig. 10c) was significantly higher in C Treated feet compared to healthy state. The heatmap demonstrates here a clear decrease in y14 denominator ASVs in the treated feet which were assigned to *Moraxellaceae, Saccharinonadaceae, Micrococcaceae, Microbacteriaceae* and *Pseudomonadaceae.*

### Differences in healthy foot microbial communities between animals that did and did not develop CODD lesions

To investigate associations between the healthy foot microbiome of the sheep and disease outcome, the differences in the microbial community diversity and composition of the feet of animals that did (case *n* = 8) or did not (control *n* = 8) go on to develop CODD lesions at the start of the study were compared using alpha and beta diversity metrics and Gneiss analysis.

**Fig. 11:**
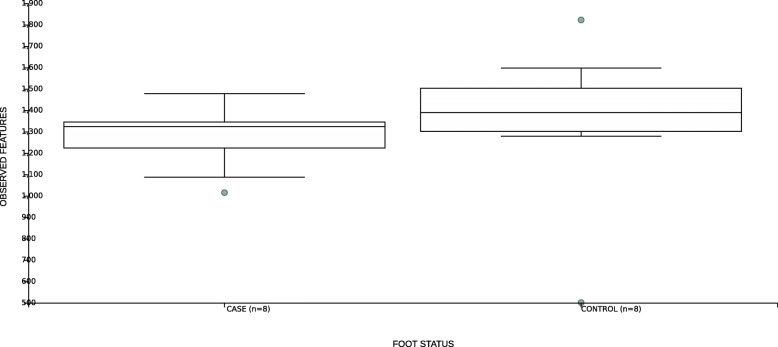
Box and whisker plot of alpha diversity as measured by observed ASVs for samples taken from healthy sheep’s feet that did (case) or did not (control) develop CODD

**Fig. 12:**
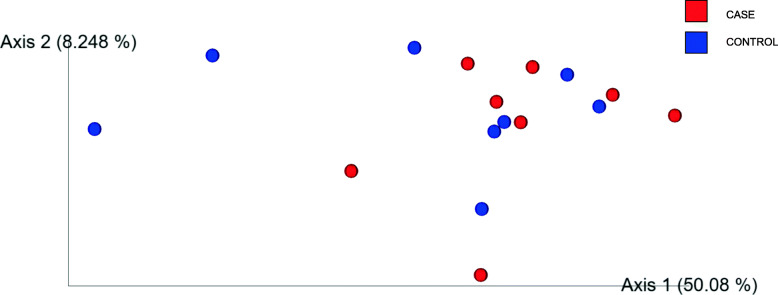
Principle Coordinate Analysis (PCoA) plot showing differences in weighted UniFrac distances of samples taken from healthy sheep’s feet that did (case) or did not (control) develop CODD in the study. Pairwise ANOSIM tests no difference between case and control samples (R test statistic = − 803, *p* = 0.803)

**Fig. 13:**
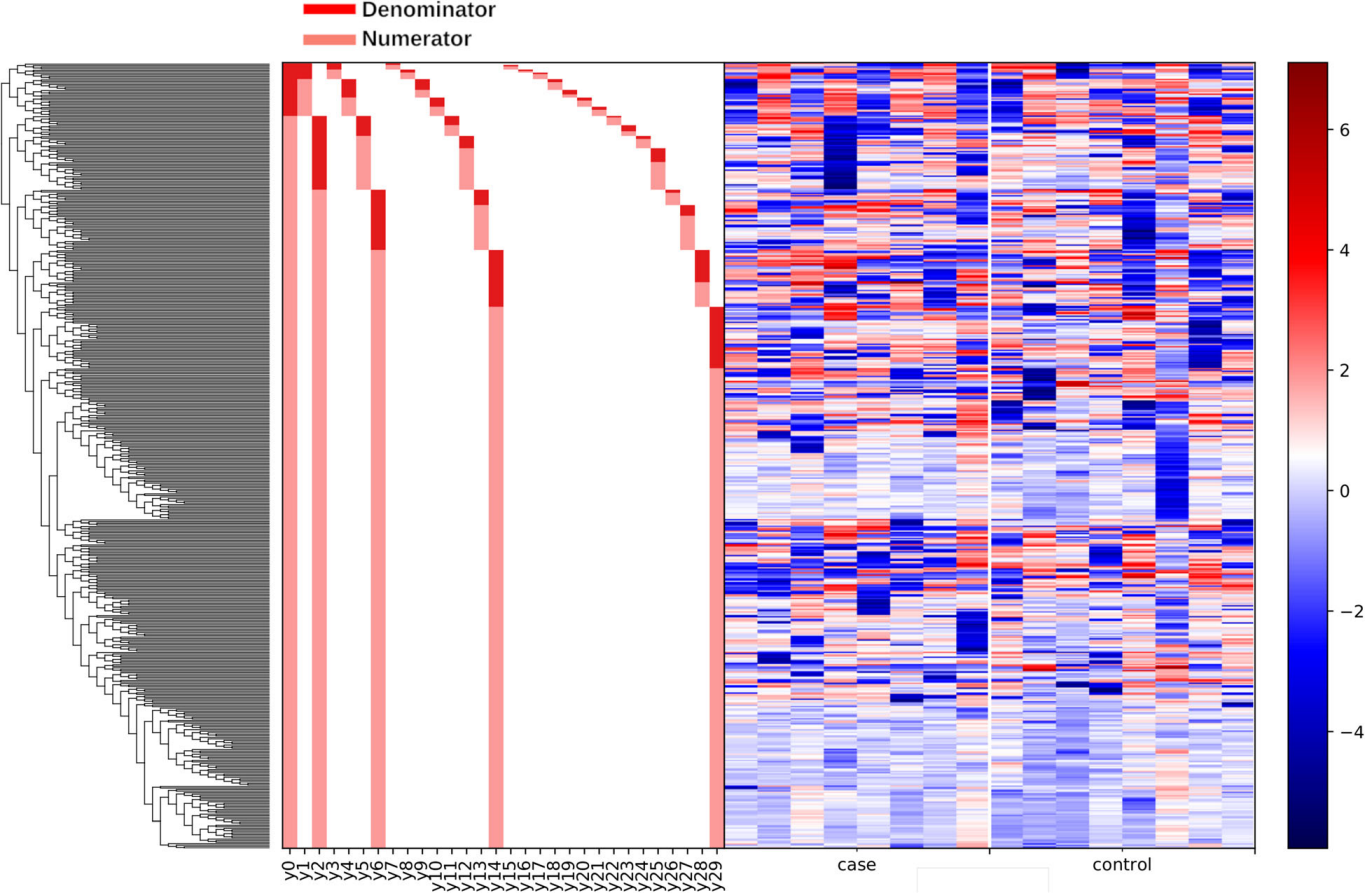
Dendogram heatmap showing log_10_ abundance of ASV in the foot microbiota of samples taken from healthy sheep’s feet that did (case) or did not (control) develop CODD in the study

Kruskall Wallis pairwise comparisons found no significant differences in ASV numbers between the foot microbiomes of the two groups (*p* = 0.207) (Fig. 11) and pairwise ANOSIM tests found no difference in the beta diversity metrics (R test statistic = − 803, *p* = 0.803) (Fig. 12). Neither the dendogram heat map (Fig. 13) or Gneiss analysis of log_10_ ratio balances (y0-y100) detected any significant microbiome compositional differences between sheep’s feet which did or did not go onto to develop CODD at the start of the study.

### Comparison of microbial taxa in CODD lesions sampled by swab and biopsy methods

**Fig. 14:**
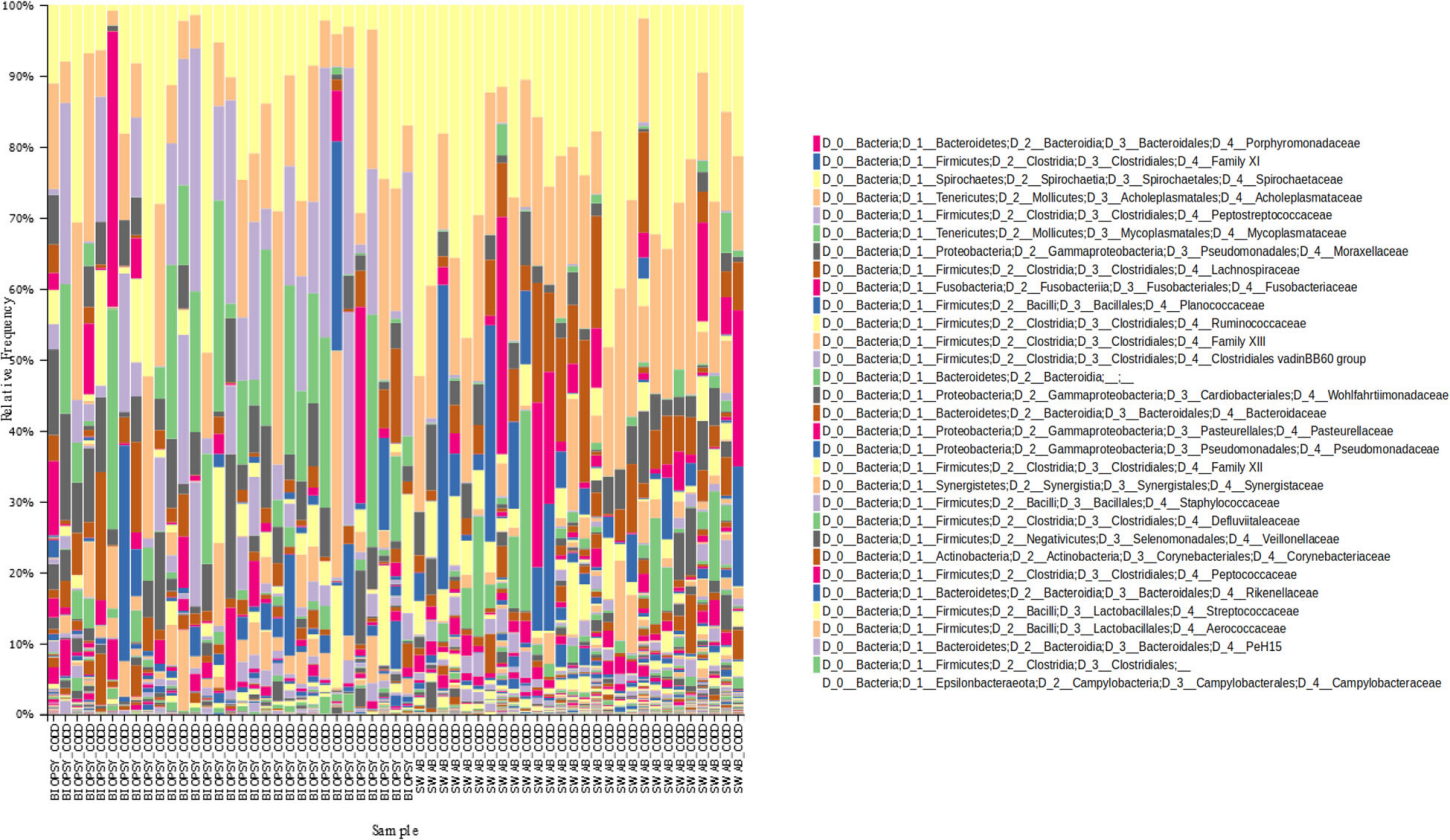
A taxa bar plot showing relative abundance of bacterial families in the microbiome of the ovine foot affected by CODD in samples obtained by lesion biopsy compared with lesion swab

The majority of studies on the microbiology of CODD have been carried out using biopsy material from CODD lesions. Whilst recognising that different sampling approaches may introduce sampling bias and thus make comparisons of results between studies difficult, repeated invasive sampling of sheep was not possible in this longitudinal experimental study. Therefore, to investigate the extent of bias caused as a result of sampling method employed, we compared the microbiomes of sheep’s feet diagnosed with clinical CODD sampled by swabbing (*n* = 28) with those sampled by biopsy (*n* = 31). From the taxa bar plots (Fig. 14), the top 15 taxa present in the two samples types were obtained. Eleven bacterial families were common to both sample types with *Porphyromonadaceae* and *Family XI* the most abundant in both sample types.

The most notable difference between the two sample types was that in the CODD swab samples there was the absence of the *Spirochaetaceae* family in the top 15 taxa; the family to which the putative causal organisms of CODD belong.

## Discussion

### Clinical description of CODD lesion development

This is the first study to describe the changes in microbial communities of the feet of sheep occurring during the development of CODD and subsequently, following treatment, to the healed state.

Previous work describing CODD has been cross sectional in nature and has described the epidemiology of the disease [14] as well as allowing development of a five point lesion scoring system to classify the clinical observations [8]. However, the cross-sectional nature of these observations means that the full disease process cannot be observed and recorded. To collect such data repeat observations of cohorts of affected animals in longitudinal studies is required. To achieve this, an experimental study to induce CODD in healthy sheep was devised by replicating on farm factors which drive infection [14]; namely, the presence of infected sheep and exposure to damp underfoot conditions (at the feed trough). We successfully induced CODD in 15 of the 30 experimental sheep. Clinically, all these sheep showed the same sequential pattern of lesion development which was from the healthy state via the development of ID lesions and thence via the development of footrot lesions to CODD lesions. The observed ID and footrot lesions were as described by [18] and the CODD lesions as described by [8].

Therefore, for this experimental study at least, the three disease states described (ID, footrot & CODD) are best considered as stages in a consistent spectrum of ovine infectious foot disease. This pattern of disease pathogenesis for CODD is surprising given that since its emergence CODD has been considered a separate disease from footrot due to its distinct appearance, failure to respond to footrot treatments [1] and proposed distinct treponemal aetiology [11, 19], although epidemiological evidence has always pointed to strong links between these diseases [14, 20]. Whilst we are confident in our observations and consider this a key paradigm in the pathogenesis of infectious foot disease, there is still a possibility that naturally occurring field cases could deviate from those observed in this experiment. However, as we did not manually abrade or macerate tissue as described in other digital dermatitis infection models [21–23] it could be considered that the circumstances of disease induction described here are the most relevant to natural disease induction in the field thus far.

### Changes in composition of sheep foot microbiome during the development of CODD

The foot microbiome demonstrated a clear pattern of reduced taxonomic diversity as the disease process progressed through each stage from healthy to ID, to footrot and CODD (Fig. 2), whilst beta diversity analysis showed distinct clustering of taxa associated with each stage of disease (Fig. 3). These findings are consistent with dominance of a reduced number of bacterial taxa which are distinct for each disease stage. Exploration of the specific taxa associated with these changes was undertaken using Gneiss analysis. The Gneiss analysis (Fig. 5), heatmap (Fig. 4), and analysis of ASVs associated with different disease states (Fig. 6 and Table 1) clearly shows a major change in the balance of microbiome composition during the disease process with *Moraxellaceae, Corynebacteriaceae, Psedomonaceae, Saccharimonadaceae, Acholeplasmataceae, Flavobacteriaceae, Ruminococcaceae, Carnobacteriaceae, Aerococcaceae, Family XI* the predominant families in the healthy feet and a progressive shift to *Porphyromonadaceae, Family XI, Bacterioida Peptostreptococcaceae, Fusobacteriaceae, Lachnospiraceae, Wohlfahrtiimonadaceae, Ruminococcaceae, Veillonellaceae, Acidaminococcaceae,* Bacteria belonging to the taxa *Porphyromonadaceae, Family XI, Veillonellaceae* and *Fusobacteriaceae* were most strongly associated with the diseased feet and their relative abundance increased progressively as the diseased worsened from healthy to ID, to footrot to CODD providing further evidence for significance in CODD pathogenesis.

*Porphyromonadaceae,* and *Fusobacteriaceae,* have all previously been associated with the pathogenesis of ovine footrot [24–27].

The family *Porphyromonadaceae* is Gram negative anaerobic family of bacteria which form part of the microbiota of the human and animal gastrointestinal tract and oral cavity. However, some species are considered to be pathogens capable of causing disease in animals and humans. Bacteria from this family have been previously cultured from ovine footrot lesions [28] associated with footrot in recent ovine foot disease microbiota studies [24, 25] where it is hypothesised that they may stimulate a change in composition of the foot microbiome as part of the aetiology of footrot. This family of bacteria have also been found in bovine foot diseases where polymicrobial aetiologies are proposed. Microbiome studies of bovine digital dermatitis [29], and claw horn and interdigital lesions [30] have identified associations between the *Porphyromonadaceae* family and diseased state, whilst in humans, *Porphyromonas gingivalis* causes severe periodontal disease [31].

The *Fusobacteriaceae* family are also Gram negative, anaerobic, non-motile and fermentative. And as with the *Porphyromonadaceae family, they are found in the oral and gastro-intestinal tract intestinal tracts of animals and humans* [32]*.* Members of this family cause a wide variety of diseases. *Fusobacterium necrophorum* is particularly associated with necrotic infections of the respiratory and gastrointestinal systems in farm animals and has long been associated with ovine footrot in where it is now considered to secondarily invade and worsen the severity of early footrot lesions caused by the primary aetiological agent., *Dichelobacter nodosus* [33]. Indeed, the *Fusobacteriaceae* family have previously been associated with the ovine footrot microbiome [26, 27]. *Fusobacterium necrophorum* is also considered to be the major aetiological agent in bovine footrot/foul in the foot together with *Bacteroides melaninogenicus* [34].

The other two families significantly associated with the diseased state in this study were *Family XI*, (a family in *Clostridiales* also known as Clostridium cluster XI) and *Veillonellaceae. Veillonellaceae* have been identified in previous microbiota sheep footrot studies ([24]. The *Veilonellaceae* are Gram-negative, anaerobic, or microaerophilic cocci and coccobacilli which can act as opportunistic pathogens in animals and humans, and are usually found in polymicrobial diseases [35]. *Clostridiales* are Gram positive spore forming anaerobes found in the soil and the gastrointestinal tract of ruminants and can be found in wounds and abscesses. Members of this family are commonly cause fatal specific disease syndromes in ruminants, however, a role for them in infectious foot disease as never previously been suggested [36].

It was surprising that although present in diseased feet samples, bacteria from the family *Spirochetaceae* were not statistically associated with the diseased foot samples (Table 1), (as collected by swabbing in the experimental study) especially as there is strong evidence [11, 12, 37] for several species of this family, *Treponema medium* phylogroup, *Treponema phagedenis* phylogroup and *Treponema pedis,* to be associated with CODD [11]. Bacteria from the treponeme genus have also been found previously in ID and footrot lesions [18, 25, 27, 38]. However, the *Spirochetaceae* family were highly abundant in the CODD biopsy samples collected in the cross-sectional farm study (Fig. 14 and Table 2), suggesting that that this family of bacteria are more readily found in deeper rather than superficial tissues and that biopsy of lesions may give a more representative picture of the dysbiosis of the microbiome associated with CODD and quite possibly other foot diseases as well. However, this invasive sampling method is only suitable for cross-sectional studies in sheep and therefore can provide only limited evidence for causality.

**Table 2:** Comparison of the 15 most abundant bacterial families present in the microbiome of the ovine foot affected by CODD in samples obtained by bacterial biopsy compared with bacterial swab

CODD SAMPLES SWAB (*n* = 24)	CODD SAMPLES BIOPSY (*n* = 31)
*Porphyromonadaceae*	*Porphyromonadaceae*
*Family XI*	*Family XI*
*Ruminicoccacae*	*Spirochaetaceae*
*Wohlfahrtiimonadaceae*	*Acholeplasmataceae*
*Moraxellaceae*	*Peptostreptococcaceae*
*Family XII*	*Mycoplasmatacea*
*Peptostreptococcaceae*	*Moraxellaceae*
*Planococcaceae*	*Lachnospiraceae*
*Lachnospiraceae*	*Fusobacteriaceae*
*Burkholderiaceae*	*Planococcaceae*
*Bacteroidaceae*	*Ruminicoccacae*
*Fusobacteriaceae*	*Family XIII*
*Synergistaceae*	*Clostridiales vadinBB60 group*
*Bacteroidales;*	*Bacteroidia*
*Corynebacteriaceae*	*Wohlfahrtiimonadaceae*

Pathogens previously identified by bacterial culture and PCR studies, as important in the pathogenesis of ID, footrot and CODD were present in Diseased foot samples. One ASV assigned to *Dichelobacter nodosus* was classified as an Intermediate ASV and was found mainly in ID samples. ASVs assigned to *Fusobacterium necrophorum* were classified as Diseased ASVs and were found mainly in ID and footrot samples. These findings are consistent with current understanding of the role of these bacteria in the pathogenesis of footrot whereby initial colonisation by *D. nodosus* is followed by secondary invasion *F. necrophorum* by [33]. Six ASVs were identified as *Treponema* with all six classified as Diseased ASVs which were present in ID and footrot samples. Three of these *Treponema* sequences were identical to those of strains from the DD associated *T. medium* phylogroup, *T. phagedenis* phylogroup and *T. pedis* ([11, 13]. Whilst a further three treponeme associated sequences are potentially also more diverse strains of *T. medium* based on current treponemal species 97% cut off [39] although caution must be applied when assigning species taxonomic classifications when using such a short region of the 16S rRNA gene. It is interesting to note given these previously DD-associated species are present on the surface in footrot and might be considered invasive within biopsies during the CODD stage that we are were observing progressive invasion starting with footrot. Indeed, future studies might benefit from attempting to differentiate whether CODD-associated treponeme presence on skin surface in footrot can predict progression to CODD development subsequently.

### Changes in the foot microbiome following treatment of CODD

All sheep affected with CODD were treated simultaneously with two doses of 10 mg/kg long acting amoxicillin (Betamox LA 150 mg/ml, Norbrook UK) given intramuscularly 48 h apart. The treatment regime chosen was based on laboratory [40] and clinical data [20] on the efficacy of amoxicllin against treponeme bacteria in CODD and FR. Two weeks post treatment all treated sheep had recovered from CODD and their lesion stage was recorded as CODD grade 5. The alpha diversity, beta diversity metrics and Gneiss analysis (of composition) (Figs. 7, 8, 9 and 10) demonstrate clearly that the microbiome of the treated sheep resembles very closely that of the healthy state of the foot at the start of the study, which is remarkable given the profound clinical and microbiological changes the feet had experienced. The principle differences between the healthy feet at the beginning compared to the post treatment feet the end of the study were in the relative abundances of families *Ruminococcaceae, Lachnospiraceae, Carnobacteriaceae, Staphylococcaceae, Pseudomonadaceae Moraxellaceae, Saccharinonadaceae, Micrococcaceae, Microbacteriaceae.* Although *Ruminococcaceae, Lachnospiraceae* and *Moraxellaceae* are found in diseased microbiomes (Table 1) none of them were identified as being significantly associated with the diseased feet microbiomes. Therefore, they may not necessarily represent persistence of pathogenic organisms in the treated feet.

### Study limitations

As with any study, the effects of sampling bias and bias induced by differences in laboratory methods must be considered when interpreting results and comparing with other studies.

During the sample collection stage, differences in microbiome data obtained could occur as a result of animal level variation or variation due to the sampling method. Both these sources of variation were explored in the study. We observed no difference in diversity or relative abundance of bacteria in the microbiota of sheep’s feet that did and did not go on to develop CODD when sampled on entry to the study (Figs. 11, 12 and 13). Importantly, as well as eliminating this as a source of bias in the results, this finding also suggests that the composition of the foot microbiota does not appear to be protective or predictive for development of CODD.

We did find important differences in the microbiota of CODD lesions depending on whether the sampling was done by lesion biopsy or swabbing (Fig. 14 and Table 2). However, it is also important to note that the populations of sheep used here for these two sample sets were different. Nonetheless the bacterial families present were broadly similar, with 11/15 families shared by both. Of particular note were the presence of families identified in this analysis study as significantly associated with the disease state; *Porphyromonadaceae, Familiy XI,* and *Fusobacteriaceae* (Table 1) which provides further evidence for the role in the pathogenesis of CODD. The finding that the *Spirochaetaceae* family (which have previously been strongly associated with CODD lesions), were more abundant in the biopsy samples, suggests that these bacteria maybe found only in deeper tissues of the disease foot and their detection is strongly influenced by sampling method. The biopsy method can only be used for single sampling of animals and not repeat sampling as required in this longitudinal study.

Other sources of bias to consider when comparing studies are the different DNA extractions methods used between studies. The method chosen here was selected to ensure extraction of DNA from treponeme bacteria. However, it is acknowledged that it is different from previous sheep foot microbiome studies [24, 25]. The V4 region of the bacterial 16S rRNA gene was chosen as it is a well-established methodology for investigating the bacterial diversity using the widely available MiSeq Illumina platform [41]. However, the use of a greater proportion of the 16S rRNA gene would be preferred as it would allow better resolution of taxa to the species level, especially given the near entire 16S rRNA gene is now widely established as the key taxonomic tool for species designation and is now approaching achievability in gene targeted metagenomics studies [42].

## Conclusions

This is the first study to report the phenotypic lesion presentation and microbiological changes in sheep’s feet during the development of CODD lesions. The principle findings of the study were:-

1. A novel pathogenesis of CODD is proposed with three distinct phenotypic lesion stages were observed in the development of CODD lesions. The initial lesion was ID which progressed to a clinical footrot lesion which developed into a CODD lesion. Therefore, better control of the initial ID and footrot lesions on farms should be prioritized to enable better CODD control.
2. Distinct microbiota were observed for each lesion stage in terms of microbial diversity, clustering and composition. *Porphyromonadaceae Familiy XI, Veillonellaceae* and *Fusobacteriaceae* were significantly associated with the diseased feet. Veillonellaceae and Fusobacteriaceae was most associated with earlier stages of CODD lesion development, namely ID and footrot stages.
3. Following treatment, the microbiota of CODD affected feet showed a strong tendency to return to the healthy state.
4. The composition of the microbiota of healthy feet did not influence the development of CODD.
5. The sampling method affected the composition of the microbiota detected in CODD lesions. However, *Porphyromonadaceae Family XI,* and *Fusobacteriaceae* families were highly abundant in both sample types providing further evidence for the role in CODD lesion development. The *Spirochaetaceae* family, although present in both, were more abundant in the biopsy samples, suggests that these bacteria maybe found more readily in deeper tissues of the disease foot.
6. It is possible using the method described to induce CODD experimentally in previously healthy sheep.

## Materials and methods

### Experimental design (longitudinal experimental study)

The project was carried out under UK Animal Scientific Procedure Act (ASPA) 1986; Home Office Project License PPL 708756 and University of Liverpool Ethics VREC417. The experimental study was supervised at all times by a Named Animal Care and Welfare Officer and a team of three veterinary surgeons. The reporting of the experiment is in accordance with the ARRIVE guidelines [43] (Supplementary file 1).

The study design was an observational study of an experimentally induced outbreak of CODD in housed sheep whereby 30 healthy, 18-month-old, Texel cross ewes were housed with 10, mixed age and breed sheep affected by CODD. Inclusion criteria for the “healthy” ewes were that they should be sourced from a single flock with no known history of CODD and they should be of the same sex, breed and age. The inclusion criteria for infected sheep were that they were sourced from farms with a history of CODD in the flock and they should have a confirmed veterinary diagnosis of an active, untreated CODD lesion in 1 foot. At the start of the study all the infected sheep were identified as positive for at least one of the hypothesised causal pathogens of CODD (*Treponema medium* phylogroup, *Treponema phagedenis* phylogroup*, Treponema pedis)* by PCR assay [13]. Sample size power calculations were not made for this study due to lack of data on the expected variation in the microbiologcal consortium; however, the sample sizes were consistent with other similar studies [44, 45]. The observational design of the experiment meant that it did not require blinding or randomizing.

Sheep were housed in a Home Office Designated Building (according to UK Animal Scientific Procedures Act, Code of Practice for Care and Accommodation of Animals) on deep litter straw bedding at a stocking rate of 1.9m^2^/sheep. Sheep were fed a maintenance ration of *ad libertum* hay. A footbath was placed under the feed racks which contained damp straw, water, and contaminated hoof clippings from a CODD infected farm to simulate naturally occurring risk factors for CODD. Sheep welfare was monitored by daily inspection of demeanour and feed intake, twice weekly mobility and body condition score and weekly veterinary clinical examination. Humane endpoints were set and if an animal reached these predetermined points (inappetence, recumbency or non-weight bearing lameness on any limb) the animal was withdrawn from study. When half of the sheep in the flock had developed CODD lesions, all sheep with any foot lesion was treated with 2 doses, 48 h apart, of a long acting amoxicillin (Betamox LA 150 mg/ml, Norbrook, Northern Ireland, UK) at dose rate of 10 mg/kg by intramuscular injection.

### Animal sampling (longitudinal experimental study)

At the start of the project and during every week of study the following data/samples were collected from each sheep, mobility score [9], body condition score [46], foot lesion score of each foot [8] and foot skin swab from each foot, regardless of disease status. If no active lesion was present the swab (Copan, Brescia, Italy) was rubbed firmly across the interdigital skin and along the dorsal border of the coronary band. When a foot lesion was present the swab was applied to the entire surface of the visible lesion. Collected swabs were immediately stored at − 80 °C until DNA extraction. Animal metadata was stored on an Access Database (Microsoft; USA).

A subset of these samples was then selected for 16S rRNA gene amplicon sequencing. To study CODD lesion development foot swab samples were selected on the basis that the foot had undergone phenotypic lesion progression from healthy state to CODD lesion. For each sheep/ foot sample set, foot swabs were then further selected in order to represent the type of clinical lesion stages observed during the development of CODD. To study microbiome changes following treatment, an additional set of samples was included for each foot which were collected from sheep’s feet 2 weeks post treatment and classed as healed (grade 5 CODD lesion [8]). In order to examine the differences in the microbiome of sheep’s feet in animals that did and those that did not go onto develop CODD, foot swab samples were selected for comparison from sheep at the start of the study (on entry to the experimental unit) and from those who remained healthy throughout the experiment and those sheep that went onto develop CODD during the experiment.

### Animal sampling (cross-sectional farm study)

To compare CODD lesions swabs with CODD biopsy material, lesion biopsy samples were collected from 31 sheep diagnosed as having CODD by a veterinary surgeon [8]. Prior to sampling, all feet were anaesthetized by infiltrating the local area with procaine hydrochloride with adrenaline (Adrenacaine, Norbrook). Prior to biopsy dry cotton swabs were drawn across the lesion. Surgical biopsies from the lesion were then obtained using a sterile 6 mm punch biopsy. After biopsy sampling all sheep were treated with antibiotics as prescribed by the farmer’s own veterinary surgeon.

### Extraction of genomic DNA

Given that we have previously established associations for DD treponemes with CODD [11] and that there are known issues surrounding detection of spirochaete DNA in microbiome studies [47, 48], we used a genomic DNA extraction methodology with a known ability to enable treponeme detection from cotton swabs. Consequently, microbial DNA was extracted from swabs using the DNeasy Blood and Tissue Kits (QIAGEN, Manchester, UK) as previously described in other studies of ruminant DD 16S rRNA gene targeted microbiome studies [49–51]. All steps were performed under sterile conditions. A negative control, not containing any sample material, was included with each extraction run. Extracted DNA samples were stored at − 80 °C until use.

### 16S rRNA gene amplification and Illumina MiSeq sequencing

The Qubit™ dsDNA HS Assay Kit (Thermo Fisher Scientific, Fair Lawn, NJ, USA) was used to measure DNA concentrations before PCR. In addition to the swab samples, ZymoBIOMICS™ Microbial Community DNA Standard was used as a mock microbial community and included as a PCR positive control. Primers [41] were used to amplify the V4 region of the 16S rRNA gene with forward primer: 5’ACACTCTTTCCCTACACGACGCTCTTCCGATCTNNNNNGTGCCAGCMGCCGCGGTAA3’.

And reverse primer: 5’GTGACTGGAGTTCAGACGTGTGCTCTTCCGATCTGGACTACHVGGGTWTCTAAT3’ [52]. Firstly, 5 μl of DNA entered a first round PCR with conditions of 20 s at 95 °C, 15 s at 65 °C, 30 s at 70^o^ C, for 15 cycles then a 5 min extension at 72 °C. Samples were purified with Ampure SPRI Beads (Beckman Coulter Genomics, Fullerton, CA, USA) before entering a second round PCR. The second PCR incorporates Illumina adapter sequences for sequencing of samples on the Illumina Sequencing platforms. Barcodes for sample identification were incorporated as follows: 16 Forward primers (i5) and 24 reverse primers (i7) each contain a separate barcode creating up to 384 different combinations. Barcode sequences were identical to those described in Illumina Nextera protocol. Generalized sequences of the forward and reverse primers are: N501f 5’AATGATACGGCGACCACCGAGATCTACACTAGATCGCACACTCTTTCCCTACACGACGCTC3’ and N701r 5’CAAGCAGAAGACGGCATACGAGATTCGCCTTAGTGACTGGAGTTCAGACGTGTGCTC3’.

Fifteen cycles of the second round PCR were performed using the same conditions as above for a total of 25 or 30 cycles. Product purification used Ampure SPRI Beads (Beckman Coulter Genomics, Fullerton, CA, USA) before being quantified by Qubit (Thermo Fisher Scientific, Fair Lawn, NJ, USA) and assessed by Fragment Analyzer (High Sensitivity NGS Fragment Analysis Kit, Advanced Analytical Technologies, Inc., Ankeny, IA, USA). Successfully generated amplicon libraries were taken forward and pooled in equimolar amounts. The quantity and quality of each pool was assessed by Bioanalyzer and subsequently by qPCR using the Illumina Library Quantification Kit (Kappa, Cape Town, South Africa) on a Roche Light Cycler LC 48011, using manufacturer’s instructions. Sample pools were sequenced on the MiSeq Illumina machine using the 2 × 300 bp chemistry. Despite showing extremely low DNA concentrations, which were not visible by agarose gel analysis, one extraction negative control was submitted for sequencing.

### Sequencing reads quality control and filtering

Raw FASTQ files were trimmed to remove Illumina adapter sequences using Cutadapt version 1.2.1 [53]. Reads were further trimmed to remove low quality bases using Sickle version 1.200 [54] with a minimum window quality score of 20. After trimming, reads shorter than 20 bp were removed.

### Amplicon sequence variant (ASV) identification and taxonomy assignment

The dada2 plugin [55] tool was used to discriminate between bases that originate from errors (either from PCR or sequencing steps) with bases that correctly correspond with the original template sequences in the denoising step and was used to create a feature table using the QIIME 2 [56] feature table plug-in. Following alignment of sequences using MAFFT [57] the phylogenetic tree was built and converted to a rooted format using the Fast Tree tool [58]. Taxonomy was assigned using the q2-feature-classifier plug-in [59] and Silva database version 132 [60]. Taxonomy profile plots were produced using the QIIME2 taxa plug-in.

### Bacterial diversity analysis

Alpha and beta diversity analyses were performed at a sampling depth of 40,000 sequences using the diversity core metrics-phylogenetic QIIME2 plug-in. This value was obtained using the diversity alpha rarefaction QIIME2 plugin and was chosen to ensure maximum sampling depth, whilst ensuring minimum sample loss (98.2% retained). Inspection of the resulting alpha rarefaction curve was used to ensure adequate sequencing depth. An observed ASV metric was used to assess species richness and compared between categories of samples using a Kruskal Wallis non-parametric test with a false discovery rate (FDR) correction. To study differences in beta diversity between groups, the rarefied abundance table was used to build pairwise sample distance matrices, using a weighted UniFrac [61] dissimilarity measure. The weighted UniFrac distance metric measures the distance between two samples considering the presence, phylogenetic distances and relative abundance of ASVs. Principal Coordinate Analysis (PCoA) was then used to visualize each distance matrix. Differences in microbiota beta diversity between groups in terms of location, dispersion or correlation structure were assessed for strength and significance using pairwise ANOSIM tests with a FDR correction [62].

### Analysis of differentially abundant amplicon sequence variants (ASV) among samples groups (gneiss analysis)

Gneiss analysis [63] was run using QIIME2 [56] to identify alteration of microbial communities with disease progression. In Gneiss analysis, shifts in the balance (ratio of abundance) between subsets of the community rather than the absolute or relative abundance of community members are calculated. The ASV table was filtered to include the most abundant 500 ASVs then the ASV abundance data log_10_ transformed and the balance calculated as the log_10_ $\frac{\mathrm{abundance}\ \mathrm{numerator}\ \mathrm{taxa}}{\mathrm{abundance}\ \mathrm{denominator}\ \mathrm{taxa}}$. The dendrogram is created using correlation clustering so ASVs which occur together more frequently in samples are grouped together on the dendrogram. Each branch represents an ASV while each node is designated a “balance” with the base node termed “y0”. The groups of ASVs on opposite branches that form a node are then designated as either the numerator or denominator. The assignment of the label “denominator” or “numerator” is arbitrary but it is consistently applied throughout the dendrogram.

Principal balances for use in Gneiss analysis were obtained via Ward’s hierarchical clustering using the correlation-clustering command producing a dendogram with 499 balances (y0-y498) created from the internal nodes of the dendogram tree. Isometric log_10_ ratios for each balance were calculated using the ilr-transform command. A multivariate response linear regression model of log_10_ ratios balances was constructed with disease status as the only covariate using the ols-regression command. Results were visualised through a regression summary and dendogram heatmaps. Balances significantly affected by the disease status were identified as those with an FDR corrected *p*-value less than 0.05. A Student’s *t*-test was used to make pairwise comparisons in balance log_10_ ratios between disease states.

Significant balances were used to classify ASVs as associated with Healthy, Intermediate or Diseased sheep. ASVs not differentially abundant (NDA) between disease states formed a fourth group. The taxonomy of ASVs identified by Gneiss analysis as more abundant in Healthy, Intermediate or Diseased sheep was examined at family level. A chi-square test was used to assess the distribution of taxa across Gneiss analysis groups. The proportion of total ASVs classified to each group (Healthy, Intermediate, Diseased, NDA) was calculated. This proportion was applied to the total number of ASVs assigned to each taxon to generate an expected number of each taxon in each group. *P*-values were corrected for multiple tests using an FDR correction. The dataset was searched for pathogens previously associated with ID, Footrot and CODD such as *Dichelobacter nodosus, Fusobacterium necrophorum* and *Treponema spp.* to identify whether they were associated with the disease state.

## Supplementary Information

**Additional file 1: Table S1.** Kruskal Wallis pairwise group comparison of alpha diversity of samples categorised by disease state and measured by observed ASV numbers. * represents *p* < 0.05.

**Additional file 2: Table S2.** ANOSIM pairwise group comparison of weighted UniFrac distances at different stages of CODD lesion development. * represents *p* < 0.05.

**Additional file 3: Table S3.** Kruskal Wallis pairwise group comparison of alpha diversity of samples from healthy (A_Healthy), CODD affected (B_CODD), antibiotic treated (C_Treated) sheep’s feet and measured by observed ASV numbers. * represents *p* < 0.05.

**Additional file 4: Table S4.** ANOSIM pairwise group comparison of weighted UniFrac distances from healthy (A_Healthy), CODD affected (B_CODD) and antibiotic treated (C_Treated) sheep’s feet. * represents *p* < 0.05.

**Additional file 5: Supplementary Document 1.** ARRIVE checklist.

**Additional file 6: Supplementary Data 2. **Reads per sample data file.

## Data Availability

The data sets generated and analysed during the study are available at https://www.ncbi.nlm.nih.gov/sra/PRJNA658364 (Accession Number PRJNA658364).

## Abbreviations

CODD: Contagious ovine digital dermatitis
FR: Footrot
ID: Interdigital dermatitis
UK: United Kingdom of Great Britain and Northern Ireland
qPCR: Quantitative polymerase chain reaction
16 S rRNA: 16 S ribosomal ribonucleic acid
DNA: Deoxyribonucleic acid
ARRIVE: Animal Research: Reporting of In Vivo Experiments
ASV: Amplicon sequence variants
PCOA: Principle component analysis
FDR: False discovery rate
ANOSIM: Analysis of similarities
