# Supplementary material for: The dysbiosis of ovine foot microbiome during the development and treatment of contagious ovine digital dermatitis
Source: Anim Microbiome. 2021 Feb 17;3:19. doi: 10.1186/s42523-021-00078-4 (PMC7888161; doi:10.1186/s42523-021-00078-4)
Supplement: Supplementary file 1 — Additional file 1: Table S1. Kruskal Wallis pairwise group comparison of alpha diversity of samples categorised by disease state and measured by observed ASV numbers. * represents p < 0.05. [file 42523_2021_78_MOESM1_ESM.docx]

| **SAMPLE CATEGORY** | **SAMPLE CATEGORY** | **H STATISTIC** | **p-VALUE** |
| --- | --- | --- | --- |
| A_HEALTHY  (n=21) | B_HEALTHY  (n=18) | 0.02401 | 0.876859 |
| A_HEALTHY  (n=21) | C_ID  (n=20) | 5.758325 | *0.023235 |
| A_HEALTHY  (n=21) | D_FOOTROT  (n=20) | 29.72014 | *4.99E-07 |
| A_HEALTHY  (n=21) | E_CODD  (n=24) | 20.91149 | *1.60E-05 |
| B_HEALTHY  (n=18) | C_ID  (n=20) | 5.539888 | *0.023235 |
| B_HEALTHY  (n=18) | D_FOOTROT  (n=20) | 27.69837 | *7.09E-07 |
| B_HEALTHY  (n=18) | E_CODD  (n=24) | 19.78359 | *2.17E-05 |
| C_ID  (n=20) | D_FOOTROT  (n=20) | 18.27148 | *3.83E-05 |
| C_ID  (n=20) | E_CODD  (n=24) | 8.000564 | *0.007794 |
| D_FOOTROT  (n=20) | E_CODD  (n=24) | 2.457325 | 0.129975 |
