# Supplementary material for: The dysbiosis of ovine foot microbiome during the development and treatment of contagious ovine digital dermatitis
Source: Anim Microbiome. 2021 Feb 17;3:19. doi: 10.1186/s42523-021-00078-4 (PMC7888161; doi:10.1186/s42523-021-00078-4)
Supplement: Supplementary file 2 — Additional file 2: Table S2. ANOSIM pairwise group comparison of weighted UniFrac distances at different stages of CODD lesion development. * represents p < 0.05. [file 42523_2021_78_MOESM2_ESM.docx]

| **SAMPLE CATEGORY** | **SAMPLE CATEGORY** | **R TEST STATISTIC** | **p-VALUE** |
| --- | --- | --- | --- |
| A_HEALTHY  (n=21) | B_HEALTHY  (n=18) | 0.314341 | *0.001 |
| A_HEALTHY  (n=21) | C_ID  (n=20) | 0.547821 | *0.001 |
| A_HEALTHY  (n=21) | D_FOOTROT  (n=20) | 0.999131 | *0.001 |
| A_HEALTHY  (n=21) | E_CODD  (n=24) | 0.879605 | *0.001 |
| B_HEALTHY  (n=18) | C_ID  (n=20) | 0.284289 | *0.001 |
| B_HEALTHY  (n=18) | D_FOOTROT  (n=20) | 0.992938 | *0.001 |
| B_HEALTHY  (n=18) | E_CODD  (n=24) | 0.748899 | *0.001 |
| C_ID  (n=20) | D_FOOTROT  (n=20) | 0.594026 | *0.001 |
| C_ID  (n=20) | E_CODD  (n=24) | 0.514494 | *0.001 |
| D_FOOTROT  (n=20) | E_CODD  (n=24) | 0.443187 | *0.001 |
