# Supplementary material for: The dysbiosis of ovine foot microbiome during the development and treatment of contagious ovine digital dermatitis
Source: Anim Microbiome. 2021 Feb 17;3:19. doi: 10.1186/s42523-021-00078-4 (PMC7888161; doi:10.1186/s42523-021-00078-4)
Supplement: Supplementary file 3 — Additional file 3: Table S3. Kruskal Wallis pairwise group comparison of alpha diversity of samples from healthy (A_Healthy), CODD affected (B_CODD), antibiotic treated (C_Treated) sheep’s feet and measured by observed ASV numbers. * represents p < 0.05. [file 42523_2021_78_MOESM3_ESM.docx]

| **SAMPLE CATEGORY** | **SAMPLE CATEGORY** | **H STATISTIC** | **p-VALUE** |
| --- | --- | --- | --- |
| A_HEALTHY  (n=4) | B_CODD  (n=21) | 8.7979765 | *0.004523 |
| A_HEALTHY  (n=4) | C_TREAT  (n=5) | 0.2400000 | 0.624206 |
| B_CODD  (n=21) | C_TREAT  (n=5) | 10.802095 | *0.003042 |
