# Supplementary material for: The dysbiosis of ovine foot microbiome during the development and treatment of contagious ovine digital dermatitis
Source: Anim Microbiome. 2021 Feb 17;3:19. doi: 10.1186/s42523-021-00078-4 (PMC7888161; doi:10.1186/s42523-021-00078-4)
Supplement: Supplementary file 4 — Additional file 4: Table S4. ANOSIM pairwise group comparison of weighted UniFrac distances from healthy (A_Healthy), CODD affected (B_CODD) and antibiotic treated (C_Treated) sheep’s feet. * represents p < 0.05. [file 42523_2021_78_MOESM4_ESM.docx]

| **SAMPLE CATEGORY** | **SAMPLE CATEGORY** | **R TEST STATISTIC** | **p-VALUE** |
| --- | --- | --- | --- |
| A_HEALTHY  (n=4) | B_CODD  (n=21) | 0.965278 | *0.0015 |
| A_HEALTHY  (n=4) | C_TREAT  (n=5) | 0.68125 | *0.019 |
| B_CODD  (n=21) | C_TREAT  (n=5) | 0.911948 | *0.0015 |

|  |
| --- |
|  |
|  |
